# Supplementary material for: Inhibitory cell populations depend on age, sex, and prior experience across a neural network for Critical Period learning
Source: Sci Rep. 2019 Dec 27;9:19867. doi: 10.1038/s41598-019-56293-2 (PMC6934704; doi:10.1038/s41598-019-56293-2)
Supplement: Supplementary file 1 — Supplementary Tables [file 41598_2019_56293_MOESM1_ESM.pdf]

Inhibitory cell populations depend on age, sex, and prior experience across a neural network for Critical Period learning

Joseph V Gogola, Elisa O Gores, Sarah E London

Supplementary tables

Tables of qualitative brain-wide expression mapping.

Table **page**

Abbreviations for neuroanatomical nomenclature used in subsequent tables ………………. 2

Whole brain GAD65 mapping ……………………………………………………………………………………. 3

Song network GAD65 mapping …………………………………………………………………………………. 4

Whole brain parvalbumin mapping ……………………………………………………………………………. 5

Song network parvalbumin mapping …………………………………………………………………………. 6

Song area cell densities and area of region analyzed ………………………………………………… 7

Telencephalon (pallium, subpallium)

HA	Apical hyperpallium
HD	Dorsal hyperpallium
M	Mesopallium
N	Nidopallium
S	Septum
A	Acropallium
E	Ectopallium
LSt (PA)	Lateral striatum
GP (PP)	Globus pallidus
SL	Lateral septal nucleus
SM	Medial septal nucleus
MSt	Medial striatum
Tn	Nucleus taeniae
B	Nucleus basalis
AL	Auditory lobule
LMAN	Magnocellular nucleus of the anterior nidopallium
Area X	Proper name
RA	Robust nucleus of the arcopallium
HVC	Proper name
Field L	Proper name
Bas	Basorostral nucleus
OM	Occipitomesencephalic tract
CDLco	Core of the dorsolateral corticoid area
Alat	Lateral acropallium

Diencephalon (thalamus/hypothalamus)

PVM (PVN)	Paraventricular nucleus
LHy	Lateral hypothalamus
GLv	Ventral part of the lateral geniculate nucleus
Rot/Rt	Nucleus rotundus
DLA	Anterior nucleus of the dorsolateral thalamus
Ov/ovco	Core of the nucleus ovoidalis
DLMco	Core of the medial part of the dorsolateral nucleus of the anterior thalamus
DP/DLP	Dorsolateral nucleus in the posterior thalamus
SpM	Medial spiriform
SpL	Lateral spiriform
EM	Ectomamillary nucleus
DMP	Posterior dorsomedial nucleus
HM/HL	Medial/lateral habenula
SPC	Parvocellular part of the superficial nucleus of the thalamus
SP/SPT	Nucleus subpretectalis
Pt	Pretectal nucleus
SubG	Subgeniculate nucleus
ALP	Posterior nucleus of ansa lenticularis
TSM	Septomesencephalic tract
OvPG	Oval pregeniculate nucleus
Ret	Reticular nucleus of the thalamus
SRt	Nucleus subrotundus
EPD	Dorsal part of the entopeduncular nucleus
VMH	Ventromedial nucleus of the hypothalamus
DMA	Dorsomedial nucleus of the anterior thalamus
DLM	dorsolateral thalamus

Mesencephalon (midbrain)

LM	Lentiform nucleus
VTA (AVT)	Ventral tegmetal area
Ico/MLd	Intercollicular nucleus/dorsal part of the lateral mesencephalic nucleus
IM/IPC	Magnocellular isthmus nuclei/parvocellular isthmus nuclei
SGC	Stratum griseum centrale
SGP/SGF	Substantia grisea et fibrosa periventricularis
LoC	Locus coeruleus
EW	Nucleus of Edinger-Westphal
Ru	Red nucleus
NIII	Nucleus of III nerve
PL/PM	Lateral/medial pons
nIV	Nucleus of IV nerve
FLM	Medial longitudinal fascicle
SN	Substantia negra
LLD	Dorsal part of the lateral lemniscal nucleus
ISo	Isthmooptic nucleus
PAG	Periaqueductal gray

Metencephalon (pons, cerebellum)

LA	Nucleus laminaris
FLM	Medial longitudinal fascicle
RPgc	Reticular nucleus of caudal pons, gigantocellularis
Dcn	Deep cerebellar nuclei
Vest	Vestibular nuclei
MC	Magnocellular nucleus
Ang	Angular nucleus
nV	Principle sensory nucleus of the trigeminal nerve
n7	Nucleus of the facial nerve

Myelencephalon (medulla)

Vest	Vestibular nuclei

GAD65

Brain Area	P25				P45				P65			
	Male		Female		Male		Female		Male		Female	
	Normal	Isolate	Normal	Isolate	Normal	Isolate	Normal	Isolate	Normal	Isolate	Normal	Isolate
HA	+(++)	+(++)	+(++)	+(++)	+(++)	+(++)	+(++)	+(++)	+(++)	+(++)	+(++)	+(++)
M	+(++)	+(++)	+(++)	+(++)	+(++)	+(++)	+(++)	+(++)	+(++)	+(++)	+(++)	+(++)
HD	+(++)	+(++)	+(++)	+(++)	+(++)	+(++)	+(++)	+(++)	+(++)	+(++)	+(++)	+(++)
N	+(++)	+(++)	+(++)	+(++)	+(++)	+(++)	+(++)	+(++)	+(++)	+(++)	+(++)	+(++)
GP (PP)	+++	++	++	++	+++	++	++	+++	+++	+++	++	+++
LSt (PA)	++	++	+(++)	+(++)	+(++)	++	+(++)	++	++	++	+(++)	+(++)
MSt	+(++)	+(++)	+(++)	+(++)	+(++)	+(++)	+(++)	+(++)	+(++)	+(++)	+(++)	+(++)
SL	+	+	+	+	+	+	+	+	+	+	+	+
SM	+	+	+	+	+	+	+	+	+	+	+	+
A	+(++)	+(++)	+(++)	+(++)	+(++)	+(++)	+(++)	+(++)	+(++)	+(++)	+(++)	+(++)
Tn	+(++)	+(++)	+(++)	+(++)	+(++)	+(++)	+(++)	+(++)	+(++)	+(++)	+(++)	+(++)
E	++	++	++	++	++	++	++	++	++	+	++	++
B	+	+	+	+	+	+	+	+	+	+	+	+
PVM (PVN)	+	+	+	+	+	+	+	+	+	++	+	+
LHy	++	+	++	++	+	++	++	+	++	++	++	++
GLv	+(++)	+	+(++)	+	+(++)	+(++)	+(++)	+(++)	+	+	++	+(++)
Rot/Rt	--	--	--	--	--	--	--	--	--	--	--	--
LA	--	--	--	--	--	--	--	--	--	--	--	--
DLA	+	++	+	+	+	+	++	+	+	+	+	+
Ov/ovco	--	--	--	--	--	--	--	--	--	--	--	--
DLMco	--	--	--	--	--	--	--	--	--	--	--	--
DP/DLP	--	--	--	--	--	--	--	--	--	--	--	--
SpM	--	--	--	--	--	--	--	--	--	--	--	--
S	+	++	++	++	+	+	+	+	+	++	+	+
SpL	++	++	++	++	++	++	+++	++	++	++	++	+
EM	+++	+	++	+	++	++	++	++	++	++	++	++
DMP	--	--	--	--	--	--	--	--	--	--	--	--
DLM	--	--	--	--	--	--	--	--	--	--	--	--
LM	++	+	+	+	+	+	++	++	++	++	+	+
VTA (AVT)	+	+	++	+	+	+	+	+	+	++	+	+
Ico/MLd	+++	++	+++	+++	+++	+++	+++	+++	+++	+++	++	+++
IM/IPC	+++	+++	+++	+++	+++	+++	+++	+++	+++	+++	+++	+++
HM/HL	--	--	--	--	--	--	--	--	--	--	--	--
SGC	+	+	+	+	+	+	+	+	++	+	+	+
SGP/SGF	++	+	++	+	++	++	+	+	++	++	+	+
SPC	--	--	--	--	--	--	--	--	--	--	--	--
SP/SPT	+++	++	+++	++	+++	++	++	++	++	++	+++	++
Pt	++	++	++	++	++	+++	+++	+++	+++	+++	++	++
EW	++	++	++	++	++	++	++	++	++	++	+	++
Ru	+	+	+	+	+	+	+	+	+	+	+	+
NIII	--	--	--	--	--	--	--	+	--	+	+	--
PL/PM	--	+	--	--	--	--	+	+	--	+	--	+
nIV	--	--	--	--	--	--	--	--	--	--	--	--
FLM	--	--	--	--	--	--	--	--	--	--	--	--
LoC	++	++	++	++	++	++	+	++	+	++	++	+
RPgc	+	+	+	+	+	+	+	+	++	+	+	+
dcn	++	+	+(++)	+	+	+	+	+(++)	+(++)	+(++)	+	+(++)
vest	++	++	++	++	++	++	++	++	+	++	++	++
AL	+(++)	+(++)	+(++)	+(++)	+(++)	+(++)	+(++)	+(++)	+(++)	+(++)	+(++)	+(++)
LMAN	+(++)	+(++)	+(++)	+(++)	+(++)	+(++)	+(++)	+(++)	+(++)	+(++)	+(++)	+(++)
Area X	+(++)	+(++)	x	x	+(++)	+(++)	x	x	+(++)	+(++)	x	x
RA	+(++)	+(++)	+(++)	+(++)	+(++)	+(++)	+(++)	+(++)	+(++)	+(+++)	+(++)	+(++)
HVC	+(++)	+(++)	+(++)	+(++)	+(+++)	+(+++)	+(++)	+(++)	+(+++)	+(+++)	+(++)	+(++)
Field L	+(++)	+(++)	+(++)	+(++)	+(++)	+(++)	++	+(++)	+	+(+++)	+(++)	++
Bas	++	++	++	++	+++	++	++	++	++	++	++	++
SN	++	++	++	+++	+++	++	++	++	++	++	++	++
MC	--	--	--	--	+	+	--	--	--	--	--	--
SubG	++	++	++	+	++	++	++	++	++	++	++	++
Ang	--	--	--	--	--	--	--	--	--	--	--	--
nV	--	+	--	--	--	--	--	--	--	--	--	--
ALP	+++	++	+++	+++	+++	++	+++	+++	+++	+++	+++	+++
LLD	++	++	++	+	++	++	+	+	+	+	++	++
TSM	--	--	--	--	--	--	--	--	--	--	--	--
OvPG	++	++	++	++	++	++	+++	++	++	++	++	++
Ret	+++	+++	+++	+++	+++	+++	+++	+++	+++	+++	+++	+++
ISo	--	--	--	--	--	--	--	--	--	--	--	--
n7	+	+	++	+	+	+	+	+	+	+	+	+
SRt	+	+	++	+	+	++	++	++	++	+	+	+
OM	+	--	+	x	--	--	--	--	x	--	x	--
CDLco	+	+	+	+	+	+	+	+	+	+	+	+
EPD	+	+	++	+	+	+	+	+	+	+	+	+
PAG	++	++	++	++	++	++	++	++	++	++	++	++
VMH	+	+	+	+	+	+	+	+	+	+	x	+
Alat	+(++)	+(++)	+(++)	+	+(++)	+(++)	+(++)	+(++)	+(++)	+(++)	+(++)	+(++)

Key	Standards
+ = majority of stained cells	+ = SM
(+) = staining on some cells in area	++ = LSt (PA)
X = area obscured	+++ = Ret

GAD65

	P25				P45				P65			
**Brain Area**	**Male**		**Female**		**Male**		**Female**		**Male**		**Female**	
	**Normal**	**Isolate**	**Normal**	**Isolate**	**Normal**	**Isolate**	**Normal**	**Isolate**	**Normal**	**Isolate**	**Normal**	**Isolate**
**AL**	+(++)	+(++)	+(++)	+(++)	+(++)	+(++)	+(++)	+(++)	+(++)	+(++)	+(++)	+(++)
**LMAN**	+(++)	+(++)	+(++)	+(++)	+(++)	+(++)	+(++)	+(++)	+(++)	+(++)	+(++)	+(++)
**Area X**	+(++)	+(++)	x	x	+(++)	+(++)	x	x	+(++)	+(++)	x	x
**RA**	+(++)	+(++)	+(++)	+(++)	+(++)	+(++)	+(++)	+(++)	+(++)	+(+++)	+(++)	+(++)
**HVC**	+(++)	+(++)	+(++)	+(++)	+(+++)	+(+++)	+(++)	+(++)	+(+++)	+(+++)	+(++)	+(++)
**Area L**	+(++)	+(++)	+(++)	+(++)	+(++)	+(++)	++	+(++)	+	+(+++)	+(++)	++

Key	Standards
+ = majority of stained cells	+ = SM
(+) = staining on some cells in area	++ = LSt (PA)
X = area obscured	+++ = Ret

Parvalbumin

Brain Area	P25				P45				P65			
	Male		Female		Male		Female		Male		Female	
	Normal	Isolate	Normal	Isolate	Normal	Isolate	Normal	Isolate	Normal	Isolate	Normal	Isolate
HA	+	+	+	+	+	+	+	+	+	+	+	+
M	+	+	+	+	+	+	+	+	+	+	+	+
HD	+	+	+	+	+	+	+	+	+	+	+	+
N	+	+	+	+	+	+	+	+	+	+	+	+
GP (PP)	++	++	++	++	++	++	++	++	++	+++	++	++
LSt (PA)	++	++	+	++	++	++	++	++	++	++	++	++
MSt	+	+	+	+	+	+	+	+	+	+	+	+
SL	--	--	--	--	--	--	--	--	--	--	--	--
SM	++	+	+	+	++	+	++	+	+	+	+	++
A	+	+	+	+	+	+	+	+	+	+	+	+
Tn	+	+	+	+	+	+	+	+	+	+	+	+
E	++	++	++	++	++	++	++	++	++	++	++	++
B	--	--	--	--	--	--	--	--	--	--	--	--
PVM (PVN)	--	--	--	--	--	--	--	--	--	--	--	--
LHy	--	--	--	--	--	--	--	--	--	--	--	--
GLv	+	+	+	+	+	+	+	+	+	+	+	+
Rot/Rt	+++	+++	+++	+++	+++	+++	+++	+++	+++	++	+++	+++
LA	--	+	+	--	+	+	+	+	+	+	--	+
DLA	+	+*	+	+	+	+	+	+	+	+	+	+
Ov/ovco	+	+	+	+	+	+	+	+	+	+	+	+
DLMco	--	--	--	--	--	--	+	--	+	+	--	+
DP/DLP	+	+	+	+	++	+	+	++	++	++	+	++
SpM	+	--	+	--	++	+	--	+	+	+	+	+
SpL	++	++	+++	++	+++	+++	+++	++	++	++	++	++
EM	+	+	+	+	++	++	++	++	++	++	+	++
DMP	--	--	--	--	--	--	--	--	--	--	--	--
LM	+	+	+	+	+	+	+	+	+	+	+	+
VTA (AVT)	--	--	--	--	--	--	--	--	--	--	--	--
Ico/MLd	+(++)	+(++)	+(++)	+(++)	+(++)	+(++)	+(++)	+(++)	+(++)	+(++)	+(++)	+(++)
IM/IPC	+++	+++	+++	+++	+++	+++	+++	+++	+++	+++	+++	+++
HM/HL	--	--	--	--	+	--	--	--	--	--	--	--
SGC	++	++	++	+	++	++	+	++	++	++	++	+
SGP/SGF	++	++	++	+	++	++	+	++	++	++	++	++
SPC	--	--	--	--	--	--	--	--	--	--	--	--
SP/SPT	+++	++	+++	++	+++	++	+++	++	+++	+++	+++	+++
Pt	+	+	+	+	+	+	+	++	+	+	+	+
EW	--	--	--	--	+	--	+	--	+	+	--	+
Ru	--	+	+	+	+	+	+	+	+	--	--	+
NIII	--	--	--	--	--	--	--	--	--	--	--	--
PL/PM	+	+	+	+	+	+	--	+	+	+	+	--
nIV	--	--	--	--	--	--	+	--	--	--	--	--
FLM	--	--	--	+	--	--	--	--	--	--	--	--
LoC	+	+*	+	++	++	++	++	++	++	+	+	++
RPgc	--	--	+	+	+	+	--	+	+	+	--	+
dcn	+	++	++	+	+	+	+	+	+	+	+	+
vest	+	+	+	+	++	+	++	++	++	+	+	++
AL	--	+	--	--	+	--	+	+	--	--	--	+
LMAN	+	+	+	--	++	+(++)	+	++	++(+++)	+(++)	+	+
Area X	+	+	x	x	++	++	x	x	++	++	x	x
RA	+	+	--	+	+++	++	--	+	+++	+++	--	--
HVC	+(++)	+(++)	--	--	+(+++)	+(+++)	--	--	++(+++)	+(+++)	--	--
Field L	--	+	--	--	+(++)	--	+	--	+	--	--	--
Bas	+(++)	+(++)	+(++)	++	+(++)	++	+(++)	+(++)	+(++)	+(++)	+(++)	+(++)
SN	+	++	++	++	+(++)	++	++	+(++)	++	++	+	+(++)
MC	+	+	++	+	++	++	+	+	++	+	+	+
SubG	--	--	+	--	--	--	--	--	--	--	--	--
Ang	++	+	++	+	++	++	++	++	++	++	++	++
nV	+	+	+	+	+	+	+	+	++	+	+	++
ALP	+	++	++	+	+	+	++	+	++	+	+	++
LLD	+	--	--	--	--	--	+	--	--	+	--	++
TSM	--	--	--	--	--	--	--	--	--	--	--	--
OvPG	+	+	+	+	+	+	+	+	+	+	+	+
Ret	+	+	+	--	+	+	--	+	+	--	+	+
ISo	--	--	--	--	--	--	--	--	--	--	--	+
n7	--	+	+	--	+	+	+	+	+	+	--	+
SRt	--	--	--	--	--	--	--	--	--	--	--	--
DLM	--	--	--	--	--	--	--	--	--	--	--	--
CDLco	+	+	+	+	+	+	+	+	+	+	+	+
EPD	--	--	--	--	--	--	--	--	--	--	--	--
Alat	+(+++)	+	+(++)	+(++)	+(++)	++(+++)	+(++)	+++	+(++)	++(+++)	++(+++)	+(++)
VMH	--	--	--	--	--	--	--	--	--	--	--	--
PAG	--	--	--	--	--	--	--	--	--	--	--	--
DMA	--	--	--	--	--	--	--	--	--	--	--	--

Key	Standards
+ = staining on majority of cells in area	+ = HA
(+) = staining on some cells in area	++ = E
X = area obscured	+++ = IM/IPC

Parvalbumin

Brain Area	P25				P45				P65			
	Male		Female		Male		Female		Male		Female	
	Normal	Isolate	Normal	Isolate	Normal	Isolate	Normal	Isolate	Normal	Isolate	Normal	Isolate
AL	--	+	--	--	+	--	+	+	--	--	--	+
LMAN	+	+	+	--	++	+(++)	+	++	++(+++)	+(++)	+	+
Area X	+	+	x	x	++	++	x	x	++	++	x	x
RA	+	+	--	+	+++	++	--	+	+++	+++	--	--
HVC	+(++)	+(++)	--	--	+(+++)	+(+++)	--	--	++(+++)	+(+++)	--	--
Field L	--	+	--	--	+(++)	--	+	--	+	--	--	--

Key	Standards
+ = staining on majority of cells in area	+ = HA
(+) = staining on some cells in area	++ = E
X = area obscured	+++ = IM/IPC

**Males**		**Count density**	**ROI area**		**Females**		**Count density**	**ROI area**
**age (Normal)**	**GAD65**	**p value**	**p value**		**age (Normal)**	**GAD65**	**p value**	**p value**
	Field L	0.11	0.91			Field L	**0.03**	0.67
	NCM	0.80	**0.03**			NCM	0.86	0.33
	CM	0.70	0.38			CM	0.51	0.36
	HVC	0.61	**0.04**			HVC	0.80	0.16
	RA	0.35	0.30			RA	0.97	0.10
	LMAN	0.52	0.99			LMAN	0.52	0.60
	Area X	0.80	0.77					
**age (Normal)**	**parvalbumin**				**age (Normal)**	**parvalbumin**		
	Field L	0.14	0.22			Field L	0.89	0.33
	NCM	0.40	0.29			NCM	0.80	0.76
	CM	0.40	0.51			CM	0.37	0.15
	HVC	0.28	0.31			HVC	0.81	0.46
	RA	**0.01**	0.08			RA	0.58	0.54
	LMAN	0.40	0.02			LMAN	0.89	0.12
	Area X	0.30	**0.04**					
**condition**	**GAD65**	**p value**	**p value**		**condition**	**GAD65**	**p value**	**p value**
	Field L	0.23	**0.02**			Field L	0.28	0.40
	NCM	0.37	0.81			NCM	0.77	0.50
	CM	0.36	0.43			CM	0.60	0.36
	HVC	0.80	0.11			HVC	0.57	0.34
	RA	0.95	0.74			RA	0.73	0.25
	LMAN	0.71	0.10			LMAN	0.71	0.68
	Area X	0.54	0.19					
**condition**	**parvalbumin**				**condition**	**parvalbumin**		
	Field L	0.15	0.09			Field L	0.93	0.33
	NCM	0.56	0.21			NCM	0.61	0.39
	CM	0.50	0.19			CM	0.15	0.85
	HVC	0.35	0.24			HVC	0.11	0.91
	RA	0.30	0.50			RA	0.51	0.60
	LMAN	0.78	0.19			LMAN	0.41	0.69
	Area X	0.72	0.16					
**age* condition**	**GAD65**	**p value**	**p value**		**age* condition**	**GAD65**	**p value**	**p value**
	Field L	0.28	0.95			Field L	**0.02**	0.09
	NCM	0.92	0.42			NCM	0.55	0.85
	CM	0.79	0.25			CM	0.19	0.20
	HVC	0.76	0.33			HVC	0.55	0.27
	RA	0.81	0.60			RA	0.93	0.29
	LMAN	0.76	0.99			LMAN	0.79	0.28
	Area X	0.61	0.54					

**age* condition**	**parvalbumin**				**age* condition**	**parvalbumin**		
	Field L	**0.01**	0.14			Field L	0.37	0.14
	NCM	0.20	0.48			NCM	0.32	0.33
	CM	0.10	0.42			CM	**0.04**	0.16
	HVC	0.90	0.90			HVC	0.19	0.96
	RA	0.68	0.71			RA	0.56	0.15
	LMAN	0.73	0.17			LMAN	0.54	0.50
	Area X	0.55	0.80					
**Males & Females**		**Count density**	**ROI area**					
**sex (Normal)**	**GAD65**	**p value**	**p value**					
	Field L	0.36	0.15					
	NCM	0.44	0.64					
	CM	0.44	0.93					
	HVC	0.08	**0.001**					
	RA	0.18	**0.007**					
	LMAN	0.83	0.74					
**sex (Normal)**	**parvalbumin**							
	Field L	0.40	0.06					
	NCM	0.86	0.82					
	CM	0.67	0.31					
	HVC	**0.02**	**<0.001**					
	RA	**<0.001**	**<0.001**					
	LMAN	0.30	0.08					
